# Evaluation of the Relationship Between Age and Trabecular Meshwork Height to Predict the Risk of Glaucoma

**DOI:** 10.1038/s41598-020-64048-7

**Published:** 2020-04-28

**Authors:** Wungrak Choi, Hyoung Won Bae, Hyuna Cho, Eun Woo Kim, Chan Yun Kim, Gong Je Seong

**Affiliations:** Institute of Vision Research, Department of Ophthalmology, Yonsei University College of Medicine, Seoul, Korea

**Keywords:** Glaucoma, Optic nerve diseases

## Abstract

This study evaluated age-related trabecular meshwork (TM) height variations in the eyes of adults in different age groups. We hypothesised that a reduction in TM occurs with increasing age. This retrospective, cross-sectional, observational study was conducted at Yonsei University Gangnam Severance Hospital between January 2015 and June 2019. We randomly included 250 eyes of 125 patients who underwent anterior segment optical coherence tomography (AS-OCT). The distance from the scleral spur to Schwalbe’s line in patients with normal open anterior chamber angle was measured using AS-OCT. Results were stratified based on patients’ age group—≤40, 41–50, 51–60, 61–70, 71–80, and 81–92 years. Thereafter, the results were compared among the age groups. The mean TM height of the patients was 770.929 ± 76.776 μm. TM height was 853.188 ± 94.117 μm in patients aged ≤40 years; it was 815.309 ± 75.723, 798.115 ± 66.040, 770.942 ± 52.774, 726.716 ± 63.979, and 715.968 ± 63.403 μm in patients aged 41–50, 51–60, 61–70, 71–80, and 81–92 years, respectively. The TM height tended to decrease with increasing age (P < 0.001). TM height was significantly shorter in older patients than in younger ones. Therefore, TM height may change with age and may contribute to increased glaucoma risk and prevalence.

## Introduction

Glaucoma is one of the most common causes of blindness worldwide^1^. Till date, it is widely accepted among glaucoma specialists that intraocular pressure (IOP) is the most important risk factor for the development of glaucoma.

Previous studies on the measurement of the trabecular meshwork (TM) size with anterior segment optical coherence tomography (AS-OCT) have been conducted^2–4^. However, the results of these studies varied substantially by study region and population ethnicity^2^. Nevertheless, the results of these studies supported the opinion that the prevalence of glaucoma may be affected by TM height. Differences in TM height could be related to differences in the incidence or risk of developing glaucoma because a shorter TM height may contribute to an increase in IOP.

It is well known that aging is closely related to increased glaucoma prevalence^5^. However, the reason for the correlation between aging and increased glaucoma prevalence is yet to be ascertained. Therefore, evaluating the relationship between age and changes in TM height may provide important clues that can clarify the association between aging and glaucoma development.

The purpose of this study was to measure the height of the TM in different age groups of patients with open anterior chamber angle and to evaluate age-related TM height variations that occur in adults. We hypothesised that the TM height becomes shorter with older age.

## Results

### Baseline characteristics

The baseline characteristics of the study population are summarised in Table 1. The mean age of the patients was 64.32 years, and the mean baseline IOP was 11.59 mmHg. The youngest participant was 32 years old and the oldest was 92 years old. The mean axial length (AXL) and central corneal thickness (CCT) were 24.45 ± 1.89 mm and 534.35 μm, respectively (Table 1).

**Table 1:** Baseline clinical characteristics.

Characteristic	Mean ± SD or N (%)
**Age [years]**	
Mean ± SD	64.32 ± 14.29
**Sex [eye]**	
Male	120 (48.78%)
Female	126 (51.22%)
Mean IOP ± SD (mmHg)	11.59 ± 3.17
Axial length ± SD (mm)	24.45 ± 1.89
CCT ± SD (µm)	534.35 ± 34.76

IOP: intraocular pressure; SD: standard deviation; CCT: central corneal thickness.

### Mean TM height categorised by age and sex

**Figure 1:**
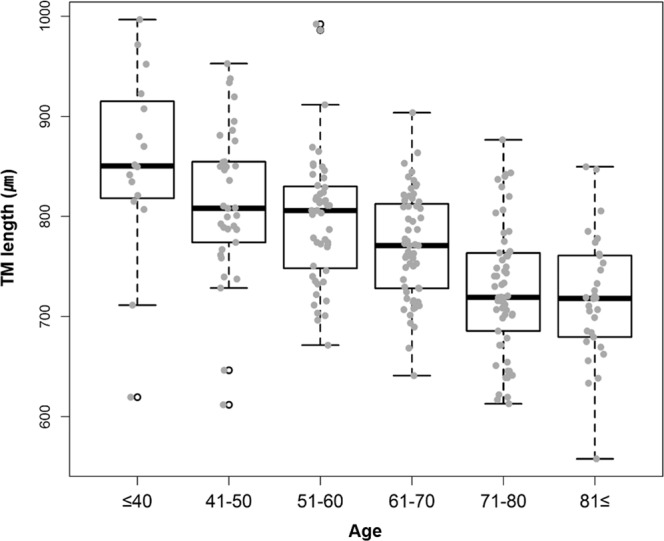
Mean trabecular meshwork height categorised by the age groups of patients. Two-hundred and forty-six eyes were included in the analysis. They were divided into six groups based on the age of the patients as follows: ≤40, 41–50, 51–60, 61–70, 71–80, and 81–92 years.

The mean TM height of the patients in our study was 770.929 ± 76.776 μm. The mean TM height among men was 764.169 ± 85.300 μm and that among women was 776.938 ± 67.120 μm; there was no significant difference between the two groups (P = 0.1911). The mean TM height of patients aged ≤40 years was 853.188 ± 94.117 μm and those of patients aged 41–50, 51–60, 61–70, 71–80, and 81–92 years were 815.309 ± 75.723 μm, 798.115 ± 66.040 μm, 770.942 ± 52.774 μm, 726.716 ± 63.979 μm, and 715.968 ± 63.403 μm, respectively. TM height tended to decrease with increasing patient age (P < 0.001). These results were validated with post-hoc analysis using the least significant difference (no correction value) and Bonferroni correction (correction value) methods (Table 2 and Fig. 1).

**Table 2:** Analysis of mean trabecular meshwork heights categorised by age group and sex.Mean trabecular meshwork height categorised by age group and sex (N = 246).

Variable	Mean ± SD (μm)			p-value		
**Age group**						
≤40 (N = 16)	853.188 ± 94.117			<0.0001		
41–50 (N = 34)	815.309 ± 75.723					
51–60 (N = 48)	798.115 ± 66.040					
61–70 (N = 60)	770.942 ± 52.774					
71–80 (N = 58)	726.716 ± 63.979					
81 ≤ (N = 30)	715.968 ± 63.403					
**Sex**						
Female	764.169 ± 85.300			0.1911		
Male	776.938 ± 67.120					
**Post-hoc analysis**						
**Post-hoc p-value - Least significant difference**						
	**≤40**	**41–50**	**51–60**	**61–70**	**71–80**	**81**≤
≤40	ref					
41–50	0.0590	ref				
51–60	0.0041	0.2453	ref			
61–70	<0.0001	0.0019	0.0341	ref		
71–80	<0.0001	<0.0001	<0.0001	0.0003	ref	
81≤	<0.0001	<0.0001	<0.0001	0.0002	0.464	ref
**Post-hoc p-value - Bonferroni correction**						
	**≤40**	**41–50**	**51–60**	**61–70**	**71–80**	**81**≤
≤40	ref					
41–50	0.8851	ref				
51–60	0.0618	>0.9999	ref			
61–70	0.0002	0.0287	0.5120	ref		
71–80	<0.0001	<0.0001	<0.0001	0.0049	ref	
81≤	<0.0001	<0.0001	<0.0001	0.0030	>0.9999	ref

Mean trabecular meshwork height was calculated and categorised by age group and sex. P value was calculated with the one-way ANOVA test (age group) and independent t-test (sex).

Post-hoc analysis was done with least significant difference (no correction value) and multiple Bonferroni correction (correction value).

IOP: Intraocular pressure; CCT: Central corneal thickness; AXL: axial length; SD: standard deviation;

### Factors that affect the TM height

In the linear regression analysis, patient age and AXL were found to be the factors that significantly affected TM height in univariable and multivariable analyses. Multivariable analysis was performed to adjust the variables with both numeric age (continuous variables, age 32 to 92) and age groups (categorical variables, age groups as ≤40, 41–50, 51–60, 61–70, 71–80, and 81–92 years). Both multivariable analysis methods showed that age had a significant influence on TM height (Table 3).

**Table 3:** Linear regression analysis - factors that affect trabecular meshwork height.

Variables	Univariable analysis		Multivariable analysis 1		Multivariable analysis 2	
	Beta (95% CI)	p-value	Beta (95% CI)	p-value	Beta (95% CI)	p-value
**Age (Numeric)**	−2.972 (−3.537–2.406)	<0.0001	−2.465 (−3.079–1.851)	<0.0001		
**Age group**						
≤40	reference				reference	
41–50	−37.879 (0.059–77.21)	0.059			−47.049 (−89.68–4.419)	0.0307
51–60	−55.073 (0.0041–92.523)	0.0041			−52.621 (−92.03–13.213)	0.0091
61–70	−82.246 (<0.0001–118.748)	<0.0001			−62.444 (−100.737–24.151)	0.0015
71–80	−126.472 (<0.0001–163.106)	<0.0001			−111.83 (−150.39–73.27)	<0.0001
81≤	−137.22(<0.0001–177.155)	<0.0001			−118.065 (−159.726–76.404)	<0.0001
**Sex**						
Female	reference		reference		reference	
Male	12.768 (−6.542–32.079)	0.194	−5.288 (−21.38–10.804)	0.518	−2.285 (−19.214–14.645)	0.7906
CCT	0.192 (−0.083–0.468)	0.1708	−0.017 (−0.267–0.234)	0.8959	−0.085 (−0.345–0.175)	0.5198
IOP	1.346 (−1.687–4.379)	0.3828	−0.556 (−3.275–2.162)	0.6871	−0.033 (−2.852–2.786)	0.9816
AXL	15.458 (10.723–20.193)	<0.0001	8.804 (4.259–13.348)	0.0002	11.078 (6.217–15.938)	<0.0001

Univariable and multivariable linear regression analyses were performed.

Multivariable analysis 1 is the result of adjusting variables with numeric age (Continuous variables) and multivariable analysis 2 is the result of adjusting variables with age groups (Categorical Variables).

IOP: Intraocular pressure; CCT: Central corneal thickness; AXL: axial length.

### Correlation analysis with TM height and variables

**Figure 2:**
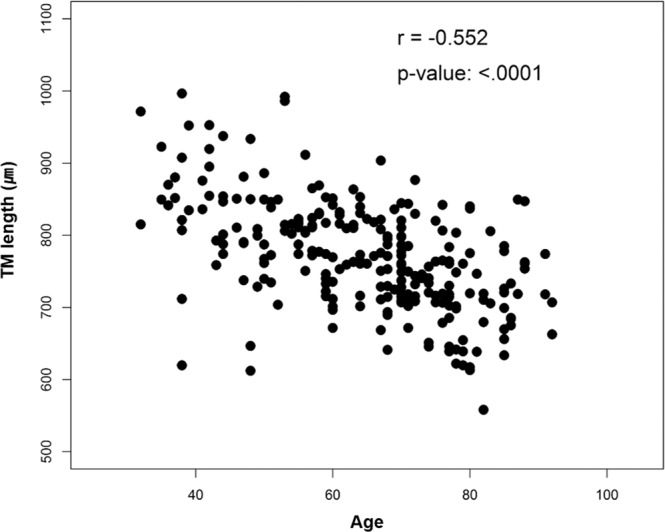
Correlation analysis with trabecular meshwork height and variables. The relationships between the trabecular meshwork height and age were evaluated using Pearson correlation coefficients (N = 246).

The relationships between TM height and age, CCT, IOP, and AXL were evaluated using Pearson correlation coefficients. Only age and AXL correlated with TM height; the correlation coefficient was −0.5517 for age (P < 0.0001) and 0.3813 for AXL (P < 0.0001) (Table 4 and Fig. 2).

**Table 4:** Correlation analysis of the relationship between trabecular meshwork height and variables.

Variables	Correlation coefficient	p-value
Age	−0.5517	<0.0001
CCT	0.0878	0.1710
IOP	0.0558	0.3832
AXL	0.3813	<0.0001

The relationship between the variables and trabecular meshwork height was evaluated using Pearson correlation coefficient.

IOP: Intraocular pressure; CCT: Central corneal thickness; AXL: axial length.

### Comparison of TM heights categorised by age

Analysis of covariance was used to compare the estimated mean TM height according to age groups after adjusting for sex, CCT, IOP, and AXL. The estimated mean TM height of the patients was 853.188 μm (±17.579) (standard error) among patients aged ≤40 years and 796.363 μm (±12.011), 790.791 μm (±9.406), 780.968 μm (±8.482), 731.582 μm (±8.524), and 725.347 μm (±11.837) among patients aged 41–50, 51–60, 61–70, 71–80, and 81–92 years, respectively.

We validated the results using post-hoc analysis with the least significant difference (no correction value) and Bonferroni correction (correction value) methods. With age ≤40 years as a reference point, all the other age groups showed significant differences in the analysis with the least significant difference method. Using Bonferroni correction, a significant difference was noted between the >60 and ≤40 years age groups (Table 5).

**Table 5:** Comparison of trabecular meshwork heights categorised by age group.

Analysis of covariance							
Variable	≤40	41–50	51–60	61–70	71–80	81≤	overall p-value
Estimated Mean TM height (SE)	843.412 (17.579)	796.363	790.791	780.968	731.582	725.347	<0.0001
**Post-hoc analysis**							
**Post-hoc p-value - Least significant difference**							
≤40	ref						
41–50	0.0307	ref					
51–60	0.0091	0.7036	ref				
61–70	0.0015	0.3197	0.4512	ref			
71–80	<0.0001	<0.0001	<0.0001	<0.0001	ref		
81≤	<0.0001	<0.0001	<0.0001	0.0001	0.6652	ref	
**Post-hoc p-value - Bonferroni correction**							
≤40	ref						
41–50	0.4602	ref					
51–60	0.1363	>0.9999	ref				
61–70	0.0225	>0.9999	>0.9999	ref			
71–80	<0.0001	0.0005	0.0001	0.0006	ref		
81≤	<0.0001	0.0008	0.0004	0.0021	>0.9999	ref	

Analysis of Covariance was used to compare the estimated mean trabecular meshwork heights of the different age groups after adjusting for sex, CCT, IOP, and AXL.

Post-hoc analysis was carried out with least significant difference (non-calibrated value) and multiple Bonferroni correction (calibrated value).

IOP: Intraocular pressure; CCT: Central corneal thickness; AXL: axial length; SE: standard error.

## Discussion

This study evaluated the height of the TM in Korean patients with open anterior chamber angle stratified into different age groups, and to the best of our knowledge, this is the first reported study to assess the same. The novel aspect of our study is that we attempted to calculate the mean TM height in different age groups of patients with open anterior chamber angle after adjusting for important variables, such as CCT and AXL.

Over the years, age-related changes in TM height have been of interest to many researchers. However, measurement of TM height became possible only recently, and it is practically difficult to determine the age-related changes in TM height that may occur in an individual because decades are needed to directly compare any changes in the height of the TM. Therefore, we randomly selected adult patients with open anterior chamber angle and compared their TM heights after categorising them into their age groups to indirectly evaluate possible changes in TM height. Because there is a known difference between the open angle and closed angle TM heights, for a more accurate comparison, we excluded patients with closed angles and performed the study only on patients with open angles^6^.

Although it is well known that aging is closely related to increased prevalence of glaucoma, the exact reason for this association has not been ascertained yet. TM height was previously reported to affect the prevalence of glaucoma^2^. This was a valuable study that put forth the idea that TM height may contribute to glaucoma development; if true, determining the TM height in patients may be helpful in assessing the risk of glaucoma development. We hypothesised that TM height may become shorter with increasing age, and this may be one of the reasons why elderly patients are more likely to develop glaucoma.

Our results showed that TM height tended to decease with increasing age. The same trend was observed with both numerical age (continuous variables) and age groups (categorical variables). This result was also validated with multivariable analysis.

There may be several reasons why TM height may decrease with aging. Firstly, it is well known that the cellularity of the TM decreases with aging; this may be one of the reasons for the decrease in TM height^7,8^. Secondly, aqueous humour production decreases with increasing age, with less volume flowing into the TM, which may result in reduced TM function, eventually leading to reduced TM size^9^. Thirdly, age-related loss of ciliary muscle movement and change in limbal corneoscleral contour may lead to reduced traction on the TM structure, resulting in decreased TM height^10–12^.

The TM structure is not only composed of TM cells but also comprises various actin-like structures, which are made of glycosaminoglycans (GAG) and extracellular matrix (ECM) proteins, such as laminin, fibronectin, and elastin. Previously, it was assumed that although TM cellularity decreases with increasing age, the amount of ECM material increases significantly with age in all regions of the TM^8^. Furthermore, the spaces corresponding to the aqueous outflow pathway of the TM also significantly decrease with increasing age^8^.

Under normal physiological conditions, even with aging, IOP remains unchanged or decreases slightly^13^. This phenomenon is attributable to the fact that the aqueous output and the amount of aqueous humour decreases with increasing age^14,15^. However, excessive changes in TM height disrupts the IOP balance, leading to the development of glaucoma.

There may be several reasons why a short TM height can increase the risk of glaucoma development. The TM is the core structure that controls IOP by regulating the outflow of aqueous humour^16^. TM function is essential for maintaining proper aqueous outflow^16^. Aqueous humour output via the TM is not just a passive pathway but is also active. Compared to a large TM, a smaller TM may have to overwork to maintain proper outflow, and subsequently, it may become less efficient than before. Moreover, a shorter TM structure may even cause the force of outflow to be weaker against the debris that can accumulate around the TM and Schlemm’s canal. Since the deposition of ECM material increases with increasing age, the accumulation of debris will be greater around a smaller TM area resulting in increased resistance to aqueous outflow.

Recently, a previous study showed that TM thickness (perpendicular to TM height) increases with age and TM height decrease was partially related with increasing age^12^. These findings were only partially consistent with our results that not all quadrants’ TM height was related with aging. This result might be because the previous study involved both children and adults (age 7–83 years) compared with our study that included only adults. However, if we exclude the 7- to 14-year data in the previous study, the results were consistent with our results that decrease in TM height was observed with increasing age.

On the other hand, the result that TM thickness increases with age was consistent with our results because as age increases, thickening of the elastic fibres of the TM and the amount of ECM increases. These changes in a patient with shortened TM height will result in increase in TM thickness. This will decrease drainage of the aqueous humour through the TM pores and conversely increase the resistance to aqueous outflow. Eventually, these changes may cause IOP elevation, although this should be studied more closely in the future. Although this aspect should be validated in future studies, a drastic change in TM height may be indicative of a future risk of glaucoma.

Notably, the results of some previously reported studies varied substantially from our results in that they did not show a trend of decrease in TM height with aging^12,17,18^. There may be several reasons for this variation. Firstly, because none of these studies were conducted on a large sample size, selection bias may have been present. Secondly, we measured TM height on the nasal and temporal sides of the eye and used the mean TM height for further analysis; previous studies measured the TM height in different quadrants and compared them separately. Finally, in our data analysis, we adjusted the data for AXL and CCT to reduce their potential influence on TM height; the previous studies did not perform such adjustment of confounding factors. This may be the most significant reason for the variation among results because we used an indirect method for the comparison of age-related changes in TM height.

The limitations of our study include its retrospective design and the indirect methodology for comparing TM heights. As it is not feasible to follow patients over decades, we utilised an indirect comparison method instead. However, follow-up and tracking of TM height changes over a long period is imperative for clarifying the exact cause of these changes. Unfortunately, this is quite difficult to accomplish in the real world. However, we performed sensitivity tests to increase the validity of our findings, and the same results were observed (Supplemental Material).

Our study has some strengths. We analysed TM height after adjusting for variables that may influence TM height. Furthermore, our sample population had a large age distribution (32–92 years). Finally, our study was conducted only on patients with an open anterior chamber angle. We believe that with these modifications, our data better reflect the real-world scenarios.

In conclusion, our study measured and evaluated the age-related changes in the TM heights among the Korean population. To the best of our knowledge, this is the first study of its kind. Our results showed that TM height was significantly shorter in older patients than in younger patients. This result may indicate that TM height can change with age, and further study is required to determine if the change in TM height contributes to increased risk and prevalence of glaucoma.

## Methods

### Ethics statement

The Gangnam Severance Hospital Institutional Review Board (IRB) approved this retrospective, cross-sectional, observational, single-centre study and provided a waiver of informed consent for the review of existing patient records (IRB number, 3–2019–0120). The methods used in this study adhered to the tenets of the Declaration of Helsinki and were Health Insurance Portability and Accountability Act compliant.

### Patient enrolment

In this study, 125 patients (250 eyes) were enrolled by randomly selecting those who underwent AS-OCT at Yonsei University Gangnam Severance Hospital, Seoul, Korea, between January 2015 and June 2019. Patients with an open anterior chamber angle who underwent AS-OCT and had records of AXL measurements were selected using a random number table. The medical records and the complete ocular examination results of these patients were systematically evaluated. Ocular examination findings included current ophthalmologic diagnosis, visual acuity, IOP, gonioscopy records, CCT, anterior chamber depth, AXL, and TM height. The exclusion criteria were as follows: history of penetrating trauma, corneal opacities that could hinder AS-OCT, closed anterior chamber angle, known cases of glaucoma, and poor quality AS-OCT images.

### Study design

Only patients with an open anterior chamber angle were included in this study. Open angle was defined as more than 90° of the posterior trabecular meshwork being visible^19^. A trained ophthalmologist measured the TM height on the nasal and temporal sides of the eye, which were manually determined to be at the 3- and 9-o’clock positions, respectively. TM height was defined as the distance between Schwalbe’s line and the scleral spur. The ophthalmologist was blinded to the baseline demographic data and ocular examination results of the patients.

The randomly selected 125 patients (250 eyes) were evaluated, and two patients (four eyes) were excluded because of poor quality AS-OCT images. Finally, 246 eyes from 123 patients were included in the analysis. The included eyes were divided into six groups based on the age of the patients—≤40, 41–50, 51–60, 61–70, 71–80, and 81–92 years. The mean TM height (mean of the nasal and temporal TM heights) was then compared among these different age groups.

### AS-OCT measurements

All patients included in this study underwent AS-OCT at Yonsei University Gangnam Severance Hospital. Anterior segment images were obtained by swept-source OCT with a CASIA AS-OCT device (Tomey Corporation, Nagoya, Japan) using the anterior angle protocol. Study participants were instructed to fixate on the centre for the scan. All measurements were performed by trained researchers who were masked to the clinical data.

Standard protocols for TM height measurement that have been described previously were used^6^. Briefly, TM height was measured in the nasal and temporal sides of the eye using AS-OCT by manually selecting the 3 and 9 o’clock positions as the nasal and temporal positions, respectively. We used one image taken in a dark-light condition along the horizontal meridian, whereas a normal-resolution scan mode was used for the results. The recently described band of extracanalicular limbal lamina (BELL) method was used to measure TM height^20^. The scleral spur was identified with the bump seen as an internal projection of the sclera into the anterior chamber, or by following the interface between the sclera and the ciliary muscle until it intersects a line projected along the inner cornea^21^. BELL was defined as a hypo-reflective band wrapping around the TM and Schlemm’s canal^20^. Location of Schwalbe’s line was defined as the corneal termination of the hypo-reflective band^20^, which was confirmed using the tip of the U-shaped interface between the cornea and the sclera^3,21^; Schwalbe’s line was assumed to be at the inner apex of the U-shape interface.

### Statistical analyses

Data were analysed by using SPSS 22.0 (IBM Corp., Armonk, NY, USA), SAS (version 9.3; SAS Institute Inc., Cary, NC, USA), and R package (version 3.2.4; http://www.R-project.org) software and are expressed as means ± standard deviations. Differences within groups were examined using the t-test, post-hoc analysis, one-way analysis of variance, analysis of covariance, and linear regression. To confirm our results, sensitivity tests were also performed by randomly selecting one eye from each individual and re-analysing the data to see if the same results would be recorded. P-values <0.05 were considered statistically significant.

### Ethics approval and consent to participate

The Gangnam Severance Hospital institutional review board approved the study and provided a waiver of informed consent for the retrospective review of existing patient records. (IRB number, 3-2019-0120). The methods used in this study adhered to the tenets of the Declaration of Helsinki and were HIPAA compliant.

## Supplementary information

Dataset 1.

## Data Availability

The datasets used and/or analysed during the current study are available from the corresponding author on reasonable request.
