# Supplementary material for: Evaluation of the Relationship Between Age and Trabecular Meshwork Height to Predict the Risk of Glaucoma
Source: Sci Rep. 2020 Apr 28;10:7115. doi: 10.1038/s41598-020-64048-7 (PMC7188680; doi:10.1038/s41598-020-64048-7)
Supplement: Supplementary file 1 — Dataset 1. [file 41598_2020_64048_MOESM1_ESM.pdf]

Evaluation of the Relationship Between Age and Trabecular Meshwork Height to Predict the Risk of Glaucoma

Wungrak Choi[1], Hyoung Won Bae[1], Hyuna Cho[1], Eun Woo Kim[1], Chan Yun Kim[1], Gong Je Seong[1*]

[1] Institute of Vision Research, Department of Ophthalmology, Yonsei University College of Medicine, Seoul, Korea

***Correspondence:** Gong Je Seong, MD

Department of Ophthalmology, Gangnam Severance Hospital, 211 Eonju-ro, Gangnam-gu, Seoul 06273, Korea

E-mail: GJSEONG@yuhs.ac

Phone: 82-2-2019-3441; Fax: 82-2-3463-1049

Supplemental data 1: Sensitivity test (N=123)

A. Sensitivity test analysis

Variables	Mean + SD (㎛)	p-value
Age group		
≤40	840.625±101.404	<.0001
41-50	812.235±80.006	
51-60	790.854±69.360	
61-70	767.617±51.852	
71-80	727.845±73.974	
81≤	720.400±39.516	
Sex		
Female	763.532±85.100	0.511
Male	772.550±65.786	

B. Pearson correlation analysis

**Variables**	**Correlation coefficient**	**p-value**
**Age**	-0.5298	<.0001
**CCT**	0.1243	0.1728
**IOP**	0.1214	0.1813
**AXL**	0.3253	0.0002

C. Post-hoc analysis

**Post-hoc p-value - Least significant difference**						
	**≤40**	**41-50**	**51-60**	**61-70**	**71-80**	**81≤**
**≤40**	ref					
**41-50**	0.3309	ref				
**51-60**	0.0748	0.322	ref			
**61-70**	0.0078	0.0322	0.2134	ref		
**71-80**	<.0001	<.0001	0.001	0.0262	ref	
**81≤**	<.0001	0.0002	0.002	0.0296	0.7306	ref

**Post-hoc p-value - Bonferroni correction**						
	**≤40**	**41-50**	**51-60**	**61-70**	**71-80**	**81≤**
**≤40**	ref					
**41-50**	>.9999	ref				
**51-60**	>.9999	>.9999	ref			
**61-70**	0.1176	0.4836	>.9999	ref		
**71-80**	0.0009	0.0013	0.0154	0.3928	ref	
**81≤**	0.0014	0.0032	0.0305	0.4447	>.9999	ref

D. Linear regression analysis - factors that affect trabecular meshwork length

Variables	Univariable analysis		Multivariable analysis 1		Multivariable analysis 2	
	Beta (95% CI)	p-value	Beta (95% CI)	p-value	Beta (95% CI)	p-value
**Age (Numeric)**	-2.817 (-3.629--2.005)	<.0001	-2.49(<.0001--3.389)	<.0001		
**Age group**						
**≤40**	Ref				ref	
**41-50**	-28.39 (0.3309--85.973)	0.3309			-45.873 (0.1685--111.459)	0.1685
**51-60**	-49.771 (-104.601-5.06)	0.0748			-56.12 (0.0682--116.503)	0.0682
**61-70**	-73.008 (0.0078--126.451)	0.0078			-65.325 (0.0285--123.645)	0.0285
**71-80**	-112.78 (-166.416--59.144)	<.0001			-109.873 (0.0003--168.267)	0.0003
**81≤**	-120.225 (<.0001--179.024)	<.0001			-112.704 (0.0006--176.319)	0.0006
**Sex**						
**Female**	ref		ref		ref	
**Male**	9.018 (0.5135--18.229)	0.5135	-5.606 (0.6406--29.332)	0.6406	-1.903 (0.8838--27.637)	0.8838
**CCT**	0.278 (-0.123-0.678)	0.1724	0.073 (0.7131--0.318)	0.7131	0.023 (0.913--0.395)	0.9130
**IOP**	2.901 (-1.367-7.168)	0.1810	-0.094 (0.9653--4.34)	0.9653	0.325 (0.8877--4.225)	0.8877
**AXL**	13.167 (6.25-20.083)	0.0003	6.89 (0.0448-0.164)	0.0448	9.403 (0.0131-2.018)	0.0131

The sensitivity test was performed by randomly selecting one eye of each individual, then the data were re-analysed to confirm the results (N=123)
A. Results of the sensitivity test, independent two sample t-test, and one-way ANOVA test were collated.
B. The relationship between the variables and mean TM length as measured with Pearson correlation coefficient
C. Post-hoc analysis was done with least significant difference (no correction value) and multiple Bonferroni correction (correction value).
D. Univariable and multivariable linear regression was performed.
Multivariable analysis 1 is the result of adjusting variables with numeric age (continuous variables) and multivariable analysis 2 is the result of adjusting variables with age groups (categorical variables)
SD: Standard deviation; IOP: Intraocular pressure; CCT: Central corneal thickness; SE: standard error; TM: trabecular meshwork; AXL: axial length

Supplemental data 2: Comparison of the trabecular meshwork lengths of different age groups

A. Analysis of covariance

Variable	**≤40**	**41-50**	**51-60**	**61-70**	**71-80**	**81≤**	overall p-value
Estimated Mean TM length (SE)	841.539 (26.688)	795.666 (18.173)	785.420 (14.089)	776.215 (12.794)	731.667 (12.789)	728.836 (18.093)	0.0005

Analysis of covariance was used to compare the estimated mean TM lengths of the age groups after adjusting for sex, CCT, IOP, and AXL.
